# A Rare Case of Disseminated Mycobacterium avium Complex Disease in a Patient Living With HIV Presenting With Endobronchial Lesions

**DOI:** 10.7759/cureus.105589

**Published:** 2026-03-21

**Authors:** Mai Motojima-Muto, Yuta Nanjo, Riko Fujioka, Kenta Izumi, Akane Hashizume, Yohei Suzuki, Yukiko Namba, Osamu Nagashima, Shinichi Sasaki, Kazuhisa Takahashi

**Affiliations:** 1 Department of Respiratory Medicine, Juntendo University Urayasu Hospital, Chiba, JPN; 2 Department of Respiratory Medicine, Juntendo University School of Medicine Graduate School of Medicine, Tokyo, JPN; 3 Department of Respiratory Medicine, Saitama Saiseikai Kawaguchi General Hospital, Saitama, JPN; 4 Department of Pathology, Juntendo University Urayasu Hospital, Chiba, JPN

**Keywords:** bronchoscopy, disseminated mycobacterium avium complex infection, endobronchial lesions, hiv, immune reconstitution inflammatory syndrome (iris)

## Abstract

Disseminated *Mycobacterium avium* complex (MAC) infection is a well-recognized opportunistic disease in patients with advanced HIV infection; however, endobronchial involvement is exceedingly rare. Here, we report a case of disseminated MAC infection presenting with progressive endobronchial lesions associated with immune reconstitution inflammatory syndrome (IRIS). A 32-year-old man living with HIV who had discontinued antiretroviral therapy (ART) presented with severe immunosuppression (CD4^+^ count: 23 cells/µL) and was diagnosed with disseminated MAC infection based on lymph-node biopsy and positive blood cultures. Antimycobacterial therapy was initiated in conjunction with ART resumption. During the treatment course, rifabutin-induced immune-mediated thrombocytopenia and amikacin-related ototoxicity occurred, necessitating multiple modifications of the treatment regimen. Despite virologic suppression and immunologic recovery, progressive left hilar lymphadenopathy and a rapidly enlarging polypoid endobronchial mass with intense fluorodeoxyglucose uptake on positron emission tomography-computed tomography developed, raising suspicion for HIV-associated malignancy. Bronchoscopic biopsy revealed granulomatous inflammation with abundant acid-fast bacilli, confirming endobronchial MAC disease consistent with IRIS. Serial bronchoscopic evaluations demonstrated gradual regression of lesions with continued antimicrobial therapy. This case illustrates the diagnostic challenge of distinguishing MAC-associated IRIS from malignancy and underscores the essential role of bronchoscopy with histopathological confirmation. Long-term, individualized management is required because clinical improvement may paradoxically coincide with airway involvement and treatment-limiting adverse events.

## Introduction

Disseminated *Mycobacterium avium* complex (MAC) infection is a critical opportunistic disease in patients with advanced acquired immunodeficiency syndrome (AIDS), particularly when CD4+ cell counts fall below 50/µL. In the era before effective antiretroviral therapy (ART) was available, the incidence of disseminated MAC was as high as 20-40%. Although the widespread adoption of ART has markedly reduced this to approximately two cases per 1,000 patients with HIV [1,2], MAC remains a significant cause of morbidity and mortality in those with profound immunosuppression.

Typical clinical manifestations of disseminated MAC include nonspecific systemic symptoms such as persistent fever, night sweats, weight loss, fatigue, and abdominal pain, often accompanied by hepatosplenomegaly and laboratory abnormalities like anemia or elevated alkaline phosphatase [2]. Although MAC frequently involves the liver, spleen, and bone marrow, endobronchial involvement is exceedingly rare. When present, endobronchial MAC lesions typically present as polypoid masses that can lead to significant airway obstruction.

Most reported cases of endobronchial MAC emerge following ART initiation as a manifestation of immune reconstitution inflammatory syndrome (IRIS) [3-6]. IRIS is categorized into two forms: paradoxical IRIS, characterized by the worsening of a previously diagnosed and treated infection, and unmasking IRIS, which is the clinical flare-up of a previously undiagnosed, occult infection following immune recovery [7,8]. These inflammatory lesions can demonstrate intense fluorodeoxyglucose (FDG) uptake on positron emission tomography-computed tomography (PET-CT) scans [6,9], closely mimicking HIV-associated malignancies such as lymphoma or Kaposi’s sarcoma. Consequently, prompt bronchoscopic evaluation with histopathological confirmation is essential to ensure an accurate diagnosis and avoid inappropriate oncological treatment.

While the rarity of endobronchial MAC-IRIS presents a diagnostic challenge, the management of such cases can be further complicated by severe treatment-related adverse events. Rifabutin is a cornerstone of MAC therapy, yet it is rarely associated with immune-mediated thrombocytopenia, a life-threatening complication. Although previous reports have described the endoscopic features of MAC-IRIS, the literature on its management is limited when standard antimycobacterial regimens are compromised by several toxicities. This report describes a unique case of endobronchial MAC presenting as unmasking IRIS, where management was significantly challenged by rifabutin-induced immune thrombocytopenia, necessitating a complex modification of the therapeutic strategy and highlighting the need for vigilant monitoring in multi-drug regimens for advanced HIV.

## Case presentation

A 26-year-old man diagnosed with HIV infection initiated combination ART consisting of elvitegravir, cobicistat, emtricitabine, and tenofovir disoproxil fumarate. He was later switched to a bictegravir-based regimen. At 30 years of age, he self-discontinued ART; at his last outpatient visit prior to discontinuation, the HIV-RNA level was 94 copies/mL (the lower limit of quantification of the assay was 20 copies/mL), and the CD4+ count was well controlled at 660 cells/µL (reference range: 500-1,000 cells/µL).

**Figure 1:**
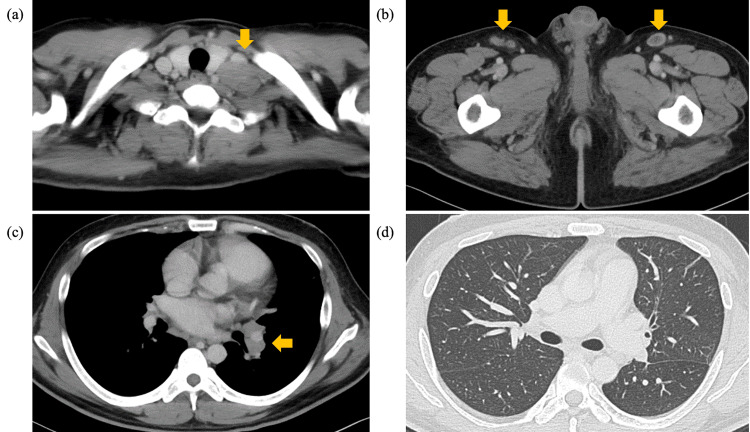
Computed tomography (CT) findings of the case on day 21. A contrast-enhanced CT scan was performed 21 days after the initial visit to our hospital. (a) Contrast-enhanced CT indicated an enlarged left supraclavicular lymph node, measuring 30 mm (yellow arrow). (b) Lymphadenopathy was observed in the bilateral inguinal regions (yellow arrows) and (c) in the left hilar region of the lung (yellow arrow). The left inguinal lymph node exhibited low attenuation internally, whereas the other lymph nodes were homogeneous, without contrast enhancement or low-attenuation areas suggestive of necrosis. Lymph nodes were visible in the axillary, mediastinal, and abdominal regions, but without definite pathological enlargement. (d) High-resolution chest CT revealed multiple small nodules randomly distributed throughout the lung fields, suggesting a hematogenous lesion.

Two years after discontinuation, at the age of 32, he developed a persistent high fever (>38ºC), prompting hospital admission. On admission, his CD4+ cell count was 23/µL, and HIV-RNA was elevated to 3.6 × 10⁶ copies/mL. Laboratory testing demonstrated marked hepatobiliary dysfunction and systemic inflammation: alkaline phosphatase, 1,460 U/L (reference range: 38-113 U/L); ferritin, 2,487 ng/mL (reference range: 30-400 U/L); lactate dehydrogenase, 467 U/L (124-222 U/L); and C-reactive protein levels, 17 mg/dL (<0.3 mg/dL) (Table 1). Contrast-enhanced CT revealed hepatosplenomegaly in addition to systemic lymphadenopathy. Specifically, an enlarged left supraclavicular lymph node (30 mm) was noted, with additional lymphadenopathy in the bilateral inguinal and left hilar regions. The left inguinal lymph node exhibited low internal attenuation, whereas other lymph nodes appeared homogeneous without necrotic features. High-resolution chest CT revealed multiple small nodules, randomly distributed throughout the lung fields, suggesting a hematogenous spread (Figure 1).

**Table 1:** Laboratory findings on day 20. Severe immunosuppression was observed (CD4+ T-cell count of 23/μL and high HIV-RNA load) alongside systemic inflammation and marked hepatobiliary dysfunction, including profoundly elevated ALP (1,460 U/L) and ferritin (2,487 ng/mL) levels. Serological testing for EBV indicates a past infection pattern. ALT, alanine aminotransferase; ALP, alkaline phosphatase; APTT, activated partial thromboplastin time; AST, aspartate aminotransferase; BUN, blood urea nitrogen; C7-HRP, cytomegalovirus antigenemia assay; Cre, creatinine; CRP, C-reactive protein; EBNA, Epstein-Barr virus nuclear antigen; EBV, Epstein-Barr virus; HIV, human immunodeficiency virus; Ig, immunoglobulin; LDH, lactate dehydrogenase; PT-INR, prothrombin time-international normalized ratio; VCA, viral capsid antigen

Parameter	Patient's Value	Reference Range	Unit
White blood cells	7,800	4,000-8,000	/µL
Hemoglobin	10.8	14.0-18.0	g/dL
Platelets	115,000	150,000-350,000	/µL
AST	153	13-33	U/L
ALT	105	8-42	U/L
ALP	1,460	38-113	U/L
LDH	467	124-222	U/L
BUN	16	8-22	mg/dL
Cre	0.57	0.61-1.04	mg/dL
CRP	17	< 0.3	mg/dL
Ferritin	2,487	30-400	ng/mL
PT-INR	1	0.85-1.15	-
APTT	62	24.3-36.0	-
CD4+ T-cell count	23	500-1,000	/µL
HIV-RNA load	3.6 × 10⁶	-	Copies/mL
EBV VCA IgG	1:160	< 1:10	-
EBV VCA IgM	< 1:10	< 1:10	-
EBV EBNA	1:20	< 1:10	-
C7-HRP	Negative	Negative	-

**Figure 2:**
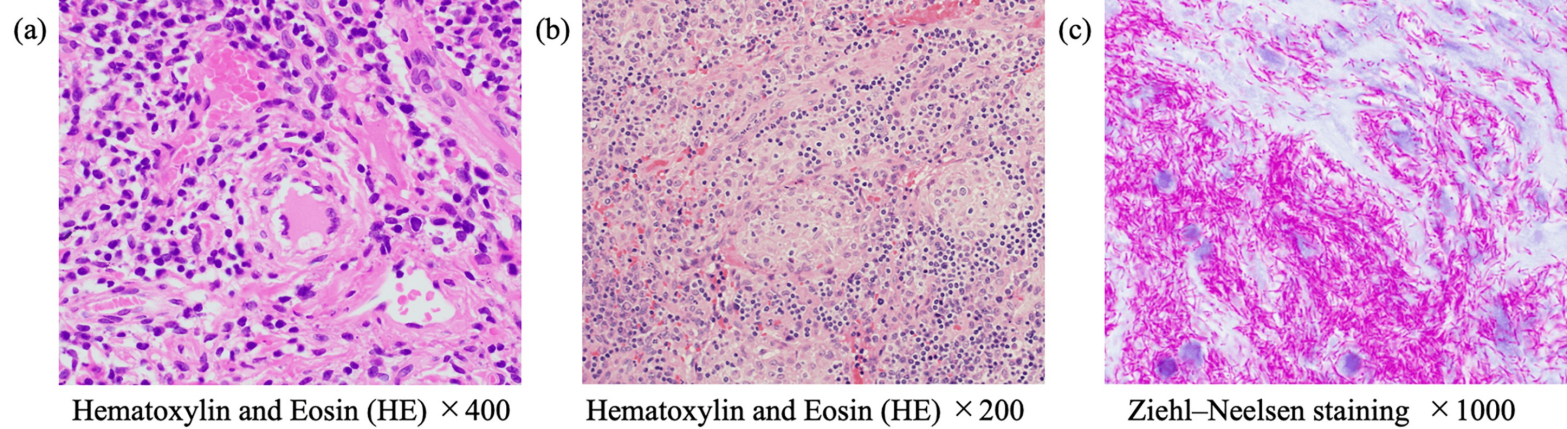
Pathological findings of the right inguinal lymph node and the left cervical lymph node. (a) A right inguinal lymph node biopsy was performed on the 28th day. Hematoxylin and eosin (HE) staining of the right inguinal lymph node (×400) indicated non-necrotizing epithelioid granulomas with Langhans-type giant cells. (b, c) Left cervical lymph node biopsy was performed on day 105. (b) HE staining of the left cervical lymph node (×200) revealed granulomas consisting of clusters of epithelioid cells containing multinucleated giant cells, accompanied by caseous necrosis. (c) Ziehl-Neelsen staining of the left cervical lymph node (×1,000) revealed numerous acid-fast bacilli.

A biopsy of the right inguinal lymph node revealed non-caseating granulomas with Langhans giant cells (Figure 2); Ziehl-Neelsen staining of the biopsy specimen was negative. To definitively rule out HIV-associated malignancies, such as lymphoma and Kaposi’s sarcoma, the specimen underwent expert hematopathology review. The evaluation confirmed the absence of atypical cells, and specific testing for lymphoma and viral markers, including Epstein-Barr virus-encoded RNA (EBER) and human herpesvirus 8 (HHV-8), was negative. Both sputum and blood cultures for acid-fast bacilli were positive for *M. avium*. The blood culture, performed using an automated liquid culture system (Mycobacteria Growth Indicator Tube (MGIT)), became positive after three weeks of incubation, whereas the sputum culture using the same MGIT system turned positive after four weeks. For both isolates, species identification was confirmed as *M. avium* through PCR analysis (Table 2). These results confirmed the diagnosis of disseminated MAC infection. Treatment was initiated with clarithromycin, ethambutol, and rifabutin. Rifampicin was avoided due to drug-drug interactions with ART, and rifabutin was selected instead. Subsequently, ART was resumed with raltegravir plus emtricitabine/tenofovir alafenamide to minimize interactions with rifabutin.

**Table 2:** Culture sample. MIC values (μg/mL) of *Mycobacterium avium* isolates obtained from various anatomical sites (blood, sputum, and left cervical lymph node) are shown across the clinical timeline. LN, lymph node; MIC, minimum inhibitory concentration

Submission Date	Day 2	Day 2	Day 109	Day 112	Day 259
Culture sample	Blood	Sputum	Blood	Left cervical LN	Sputum
Bacterial collection fluorescence method	Negative	Negative	Negative	2+	1+
Culture period (weeks)	3	4	4	2	4
Antibiotics	MIC (mdg/mL)	-	-	-	-
Kanamycin	2	4	4	8	4
Amikacin	2	4	2	4	4
Clarithromycin	0.12	0.25	0.12	0.25	0.25
Levofloxacin	0.5	0.5	0.5	0.5	0.5
Streptomycin	2	2	2	4	2
Rifampicin	0.25	0.5	0.25	1	0.5
Ethambutol	4	4	4	4	4
Rifabutin	0.12	0.12	0.12	0.12	0.12

Twelve days after initiating treatment with clarithromycin, ethambutol, and rifabutin, the patient developed severe thrombocytopenia, with platelet counts dropping from 200,000/µL to 6,000/µL (reference range: 153,000-346,000/µL), despite the absence of bleeding symptoms. Platelet transfusions were ineffective, and platelet-associated IgG was elevated at 1,530 ng/10⁷ cells (reference range: under 46 ng/10⁷ cells). All oral medications were discontinued, but thrombocytopenia persisted. We suspected an immune-mediated mechanism, and prednisolone was initiated at 70 mg/day (1 mg/kg), which resulted in gradual platelet recovery. Nine days after corticosteroid initiation, platelet count improved to approximately 100,000/µL. Antimycobacterial treatment was resumed two weeks after discontinuation. Given that the thrombocytopenia was likely induced by rifabutin, the drug was replaced with levofloxacin. This choice was based on susceptibility testing and National Institutes of Health/Centers for Disease Control and Prevention guidelines, which suggest adding a fourth agent for severe disease characterized by CD4+ cell counts less than 50 cells/µL or high mycobacterial loads [2]. This intensified regimen, including clarithromycin and ethambutol, led to stabilized platelet counts, returning to approximately 200,000/µL. Prednisolone was tapered over 71 days without recurrence of thrombocytopenia. The dosage was tapered as follows: 70 mg/day for nine days, 50 mg/day for five days, 30 mg/day for 42 days, 15 mg/day for nine days, 10 mg/day for four days, and 5 mg/day for two days.

**Figure 3:**
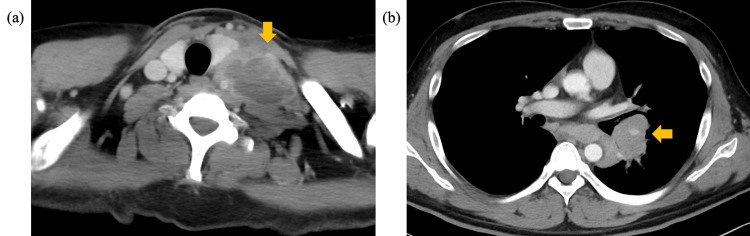
Computed tomography (CT) of the left cervical lymph node and the left hilar lymph node. (a) Contrast-enhanced CT on day 112; the left supraclavicular lymph node was enlarged (yellow arrow). (b) Contrast-enhanced CT on day 176; enlarged left hilar lymph nodes were identified (yellow arrow).

Three months after the initiation of treatment, the CD4+ cell count increased to approximately 50/µL, and HIV-RNA dropped below 10² copies/mL, indicating a favorable virologic response. Despite therapy, enlargement of the left supraclavicular lymph node was observed when the prednisolone dose was tapered to 30 mg, and biopsy revealed numerous acid-fast bacilli (Figures 2-3). Tissue culture grew *M. avium* after three weeks of incubation, and the isolate remained fully susceptible to clarithromycin (Table 2). Intravenous amikacin was added to the regimen. The dose was appropriately weight-adjusted, initiated at 1,200 mg/day (15 mg/kg). Given the persistent airway involvement and the high mortality risk associated with disseminated MAC in this patient, amikacin was added to the regimen [2]. Therapeutic drug monitoring was routinely performed, and the trough concentrations of amikacin were maintained between approximately 1.0 and 2.4 μg/mL to minimize the risk of toxicity. Although formal audiometry was not performed prior to the initiation of amikacin, the patient remained free of subjective auditory or vestibular symptoms throughout the treatment course.

**Figure 4:**
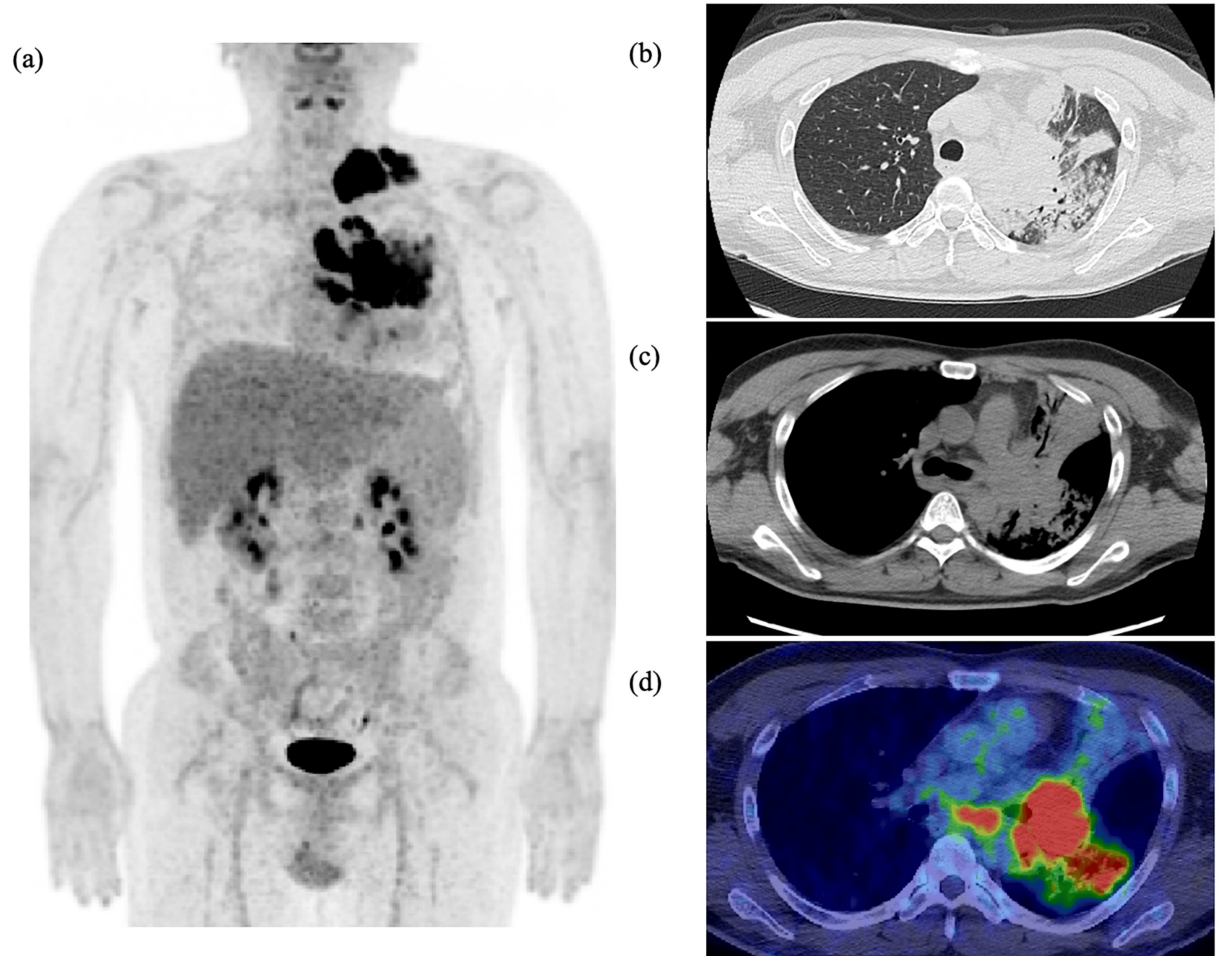
Positron emission tomography-computed tomography (PET-CT) findings of the case. (a) PET-CT on day 208. (b, c) There is a large infiltrative shadow extending from the left upper lobe to the lower lobe. This area shows high uptake with an SUVmax of 17. (d) Additionally, multiple lymphadenopathies are observed at the left hilum, left paratracheal region, left supraclavicular, and left infraclavicular areas, all demonstrating high uptake.

**Figure 5:**
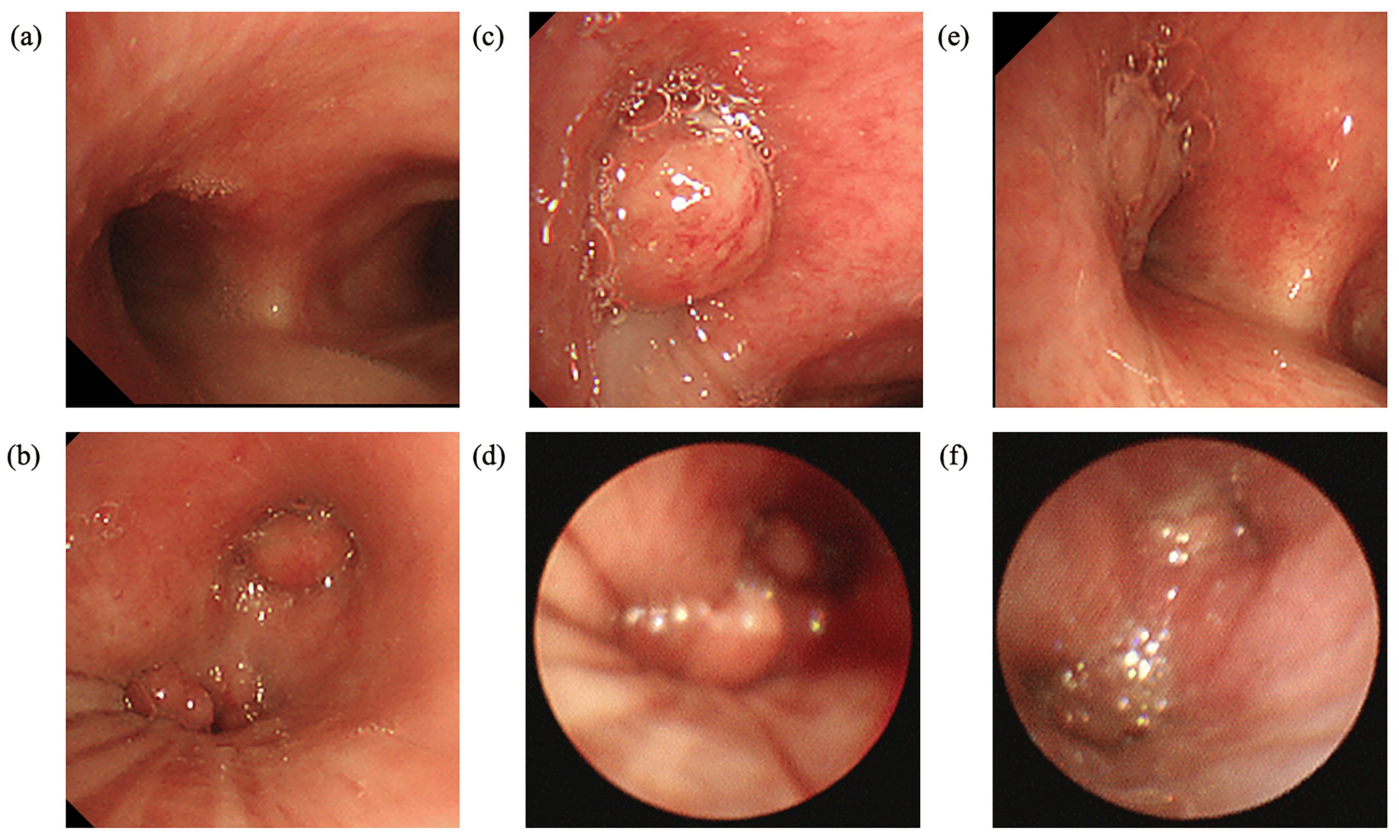
Bronchoscopy findings of the case. (a, b) Bronchoscopy on day 200 (BF 1). (a) Mild erythema was observed at the entrance of the left main bronchus, but no granulomatous lesions were apparent. (b) Granulomatous lesions were observed at the entrances of both the left upper and lower lobe bronchi. A biopsy was performed at the entrance of the left lower lobe bronchus. No ulceration, bleeding, or pulsation was noted. (c, d) Bronchoscopy on day 300 (BF 2): (c) A new granulomatous lesion was evident at the entrance of the left main bronchus. (d) Bronchial lumens were re-evaluated using a 4-mm scope. Granulomatous lesions persisted at the entrances of both the left upper and lower lobe bronchi, with mild enlargement at the lesion in the left lower lobe bronchus. (e, f) Bronchoscopy on day 407 (BF 3): (e) Granulomatous lesion at the entrance of the left main bronchus exhibited signs of regression. (f) Lesions at the entrances of the left upper and lower lobe bronchi were again visualized with a 4-mm scope, and both demonstrated a trend toward resolution.

**Figure 6:**
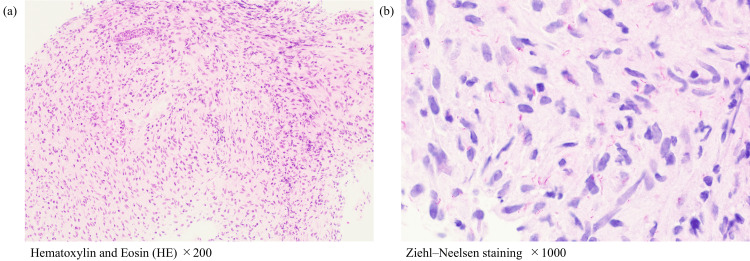
Histopathological findings of the case. (a, b) Histopathological findings of the endobronchial lesion on day 201. (a) Hematoxylin and eosin staining (×200) indicated granulomatous inflammation with epithelioid histiocytes and spindle-shaped nuclei. (b) Ziehl-Neelsen staining (×1,000) revealed numerous acid-fast bacilli within the granuloma.

Six months after initiating treatment, the patient developed exertional dyspnea and expiratory wheezing at rest. Imaging revealed progressive enlargement of the left hilar lymph node, resulting in extrinsic compression of the left main bronchus (Figure 3). Although the pulmonary parenchymal lesions had remained stable during corticosteroid therapy, significant radiological progression was observed following the recovery of the CD4+ count to approximately 100/µL, whereas the HIV-RNA viral load remained suppressed at less than 100 copies/mL. Drug susceptibility testing was performed for the *M. avium* isolates recovered during the clinical course (Table 1). The minimum inhibitory concentrations (MICs) for clarithromycin remained consistently low (0.12-0.25 μg/mL), confirming the absence of acquired macrolide resistance. Given the atypical clinical course, the differential diagnosis was broadened. Serum soluble IL-2 receptor (sIL-2R) levels were mildly elevated at 956 U/mL (reference range: 157-474 U/mL), whereas tumor markers, including those for lung cancer, remained within normal limits. Although the initial inguinal lymph node biopsy performed six months prior had rigorously ruled out lymphoma and Kaposi’s sarcoma (demonstrating negative EBER and HHV-8), the possibility of a newly developed HIV-associated malignancy was reconsidered. To further evaluate for possible malignancy, PET-CT was performed, revealing a large infiltrative shadow extending from the left upper lobe to the lower lobe, with high uptake (SUVmax, 17; Figure 4). Additionally, multiple lymphadenopathies were observed at the left hilum, left paratracheal region, and the left supra- and infraclavicular areas, all demonstrating similarly high uptake. Bronchoscopy was performed for diagnostic reassessment and revealed a broad-based, polypoid endobronchial mass at the orifice of the left upper and lower lobes with mildly erythematous and smooth mucosa. This lesion corresponded to the area showing FDG uptake on PET-CT. The mass partially compressed the bronchial lumen, but airway patency was preserved (Figure 5). Histopathology demonstrated granulomatous inflammation with abundant acid-fast bacilli, consistent with endobronchial MAC disease (Figure 6). Considering that the clinical course was consistent with IRIS-related worsening of disseminated MAC, continuation of the existing antimicrobial regimen was deemed appropriate.

**Figure 7:**
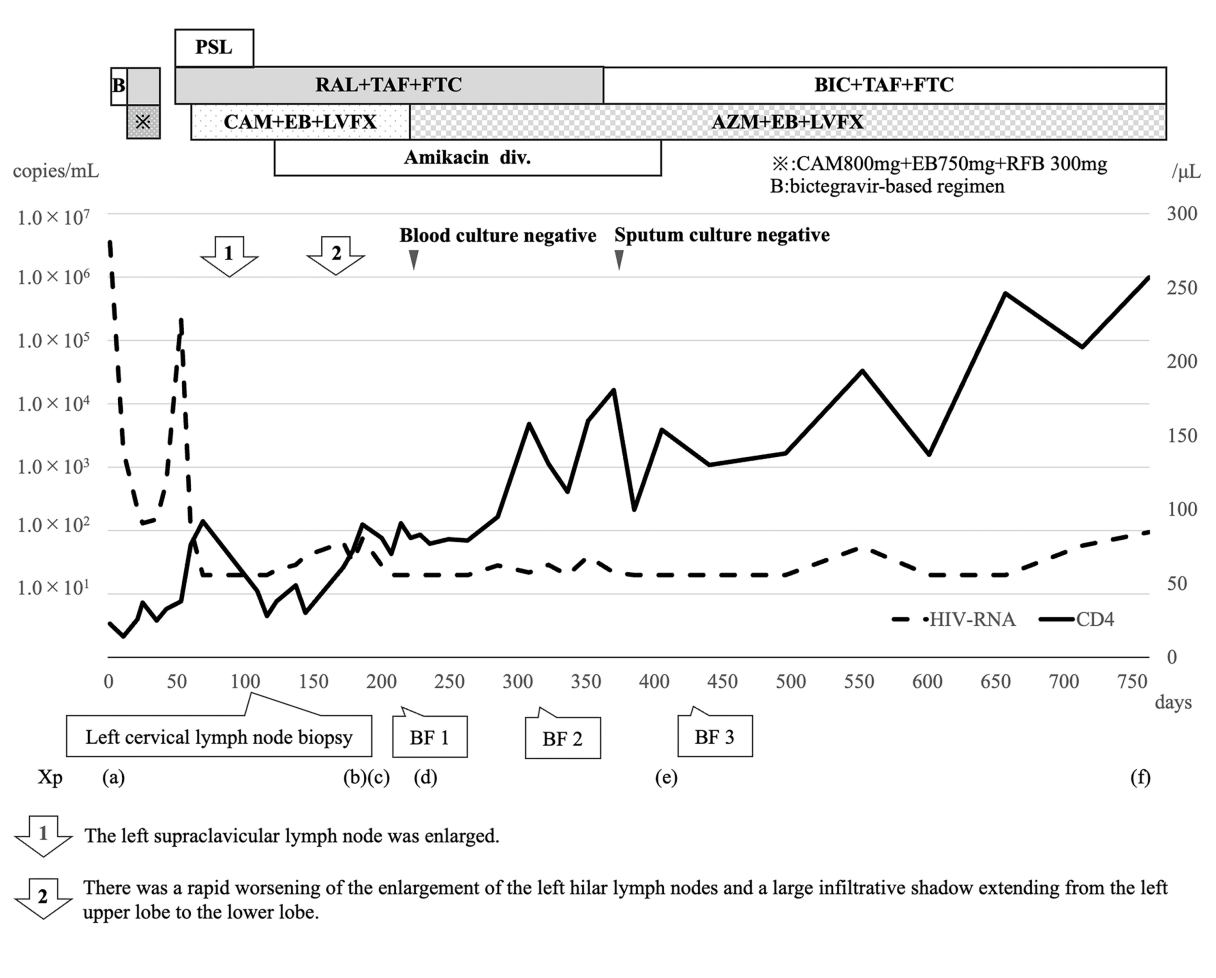
Treatment course and chest radiography findings of the patient. Clinical course of the patient showing changes in plasma HIV-RNA levels (log scale) and CD4+ T-cell counts over time in conjunction with antimycobacterial and antiretroviral therapies. The lower detection limit for HIV-RNA was 20 copies/mL; values below this are shown as 20. Antiretroviral therapy (ART) was initially started with a bictegravir (BIC)-based regimen. Because of potential drug-drug interactions with rifabutin, the regimen was changed to raltegravir (RAL), tenofovir alafenamide (TAF), and emtricitabine (FTC). Thereafter, the patient was switched back to a BIC-based regimen from ART. Antimycobacterial therapy: Initial therapy comprised clarithromycin 800 mg/day, ethambutol 750 mg/day, and rifabutin 300 mg/day (see ※). Due to rifabutin-induced thrombocytopenia, rifabutin was replaced with levofloxacin 500 mg/day. Clarithromycin was subsequently discontinued because of diarrhea, and the regimen was modified to azithromycin 600 mg/day, ethambutol 750 mg/day, and levofloxacin 500 mg/day. Intravenous amikacin (1200 mg/day) was temporarily added during the period indicated. Clinical and radiological events: (1) Enlargement of the left supraclavicular lymph node was observed. (2) Rapid radiological worsening occurred, characterized by significant enlargement of the left hilar lymph nodes and a large infiltrative shadow extending from the left upper lobe to the lower lobe. Corticosteroid therapy: Prednisolone (PSL) was administered from day 51 to 121 for the treatment of thrombocytopenia. The dosage was tapered as follows: 70 mg/day for nine days, 50 mg/day for five days, 30 mg/day for 42 days, 15 mg/day for nine days, 10 mg/day for four days, and 5 mg/day for two days. Bronchoscopy findings (BF 1-3): The three timepoints at which endobronchial lesions were evaluated via bronchoscopy. Image made using Microsoft PowerPoint (Microsoft® Corp., Redmond, WA).

**Figure 8:**
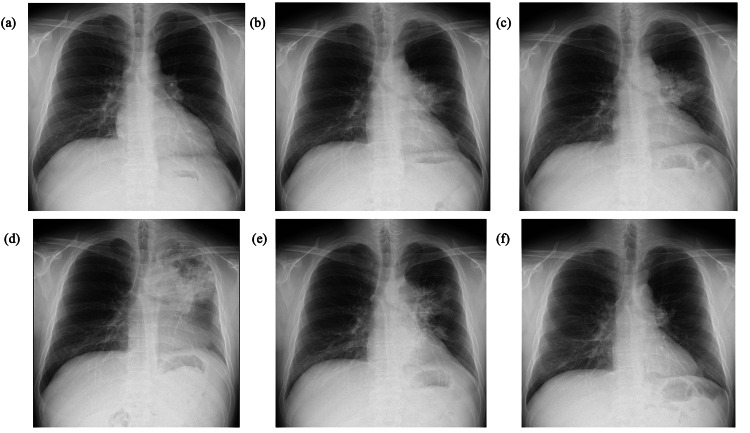
The treatment course and chest radiography findings of the patient. (a) Chest radiography at the first visit indicated mediastinal lymphadenopathy. (b) On day 188, a new infiltrative shadow was evident in the left upper lung field. (c) On day 199, the infiltrative shadow in the left upper lung field had worsened. (d) On day 227, the infiltrative shadow showed further progression. (e) On day 406, the infiltrative shadow showed signs of improvement. (f) Observations on day 761 indicated that the infiltrative shadow had resolved, but mild mediastinal lymph node swelling remained.

At approximately seven months after the initiation of therapy, mycobacterial blood cultures converted to negative, and the endobronchial lesion showed gradual regression; however, sputum cultures remained positive for *M. avium*. Regarding pulmonary lesions, small nodular shadows in both lungs had disappeared. Localized infiltrative shadows were observed at the peripheral side of the airway lesions associated with obstruction in the left lung, and nodular shadows were observed extending from the bronchi. Despite the isolate’s susceptibility to clarithromycin, the treatment was switched to a regimen of azithromycin, ethambutol, levofloxacin, and amikacin due to clarithromycin-induced diarrhea. A repeat bronchoscopy performed at 10 months revealed a newly developed polypoid lesion at the entrance of the left main bronchus, whereas the previously observed polypoid lesion had slightly decreased in size (Figure 5). Given that the patient’s airway symptoms were improving, the current antimicrobial regimen was continued. Subsequently, his CD4+ cell count persistently exceeded 100/µL, and chest radiography demonstrated improvement of the infiltrative shadow. A follow-up bronchoscopy at approximately 13 months revealed a reduction in the polypoid lesions involving both the main bronchus and the endobronchial bronchi (Figure 5). These morphological improvements were paralleled by clinical recovery, with resolution of dyspnea and wheezing. However, hearing loss at 4,000 and 8,000 Hz developed, likely due to amikacin-induced ototoxicity. Amikacin therapy was discontinued after 10 months of administration. The patient received a multidrug regimen consisting of azithromycin, ethambutol, and levofloxacin. The endobronchial lesions and multiple lymphadenopathies showed progressive improvement as the CD4+ count consistently exceeded 100/µL (Figures 7-8). One year after the initiation of treatment, the sputum culture test was negative. The patient continued this regimen at our institution for approximately two years. Although long-term maintenance therapy was planned, the patient was referred to another hospital due to relocation. At the time of transfer, the CD4+ count had recovered to 257/µL and the HIV-RNA viral load was suppressed to 95 copies/mL, confirming sustained microbiological negativity and successful immune reconstitution.

## Discussion

Endobronchial MAC lesions are rare, even among immunocompromised patients, with only a few reports documenting their bronchoscopic findings [3-6]. Among these, only one case strictly met the standardized criteria for paradoxical IRIS [7]. Most other reported cases likely represented “unmasking” IRIS, as MAC was first diagnosed after ART initiation. Similarly, our patient had asymptomatic disseminated MAC at the time of ART resumption, with blood cultures subsequently confirming the infection, characterizing this as a case of unmasking MAC-IRIS.

The diagnosis of IRIS in this case was established based on the criteria proposed by French et al. [8]. Specifically, the patient met two major criteria: (A) an atypical inflammatory presentation, characterized by the progressive enlargement of the hilar lymph node and development of endobronchial lesions despite ongoing pathogen-specific therapy, and (B) a significant decrease in plasma HIV-RNA concentration to below 100 copies/mL. Additionally, the patient met the minor criterion of a sustained increase in the CD4+T-cell count following the resumption of ART. The clinical course highlights a complex interplay between corticosteroid therapy, mycobacterial proliferation, and IRIS. The progression of lymphadenopathy occurred during the prednisolone taper, and the biopsy revealed numerous acid-fast bacilli on Ziehl-Neelsen staining. Although the presence of abundant bacilli might suggest treatment failure, we interpret this as a dual pathological process. Initially, high-dose corticosteroid therapy likely facilitated microbial proliferation by suppressing the early inflammatory response, leading to a high antigen burden. As the immunosuppressive effect of the steroids waned and ART-induced immune reconstitution progressed, the restored immune system reacted intensely to these persistent antigens, transitioning to IRIS-associated inflammatory expansion. Several factors further stand contrary to alternative explanations, such as antimicrobial resistance or inadequate drug penetration. Serial susceptibility testing confirmed that the *M. avium* isolate maintained high susceptibility to clarithromycin (MIC 0.12-0.25 µg/mL). Regarding drug delivery, although tissue concentrations were not directly measured, the conversion of blood cultures indicated effective systemic distribution. Given that the lesions eventually resolved without switching to a different class of antimicrobials, the clinical decline was clearly attributable to an IRIS-related flare-up following immunological recovery rather than a lack of drug efficacy.

In previously reported cases of disseminated MAC disease, bronchoscopic findings have consistently shown polypoid lesions. Although most cases in immunocompetent patients have been reported to be caused by *M. intracellulare*, they also exhibited a similar polypoid morphology [10,11]. Pathological findings in both HIV-infected and non-HIV cases have revealed macrophage-dominant inflammatory changes, suggesting that an incomplete immune response to MAC infection may contribute to polyp formation within the bronchial lumen [3-5,10,11].

Intratracheal involvement due to disseminated MAC is often difficult to distinguish from malignancies and Kaposi’s sarcoma based on imaging alone, especially when the lesions show increased uptake on PET-CT, thereby mimicking malignant tumors. In MAC infection, activated macrophages play a central role in granuloma formation, which likely explains the marked FDG uptake observed in MAC-related granulomatous lesions. Furthermore, FDG uptake on PET-CT has been reported to decrease in parallel with clinical improvement, suggesting that PET-CT may also be useful for assessing treatment response [5,9]. Given that treatment for disseminated MAC infection often extends from several months to more than one year, long-term radiological follow-up is required [3-6]. In the present case, serial PET-CT imaging was not performed; however, treatment response was monitored using serial chest CT scans. These scans confirmed marked regression of the mediastinal lymphadenopathy and endobronchial lesions, correlating with the patient’s clinical improvement.

Bronchoscopy is especially useful in differentiating disseminated MAC infection from pulmonary Kaposi’s sarcoma. Bronchoscopic findings in pulmonary Kaposi’s sarcoma are typically characterized by bright red, oval, elevated mucosal lesions, and pathological findings, including the presence of spindle-shaped cells, are definitive for diagnostic differentiation [12-14]. Therefore, a combination of bronchoscopic evaluation, histopathological examination, and microbiological analysis is essential to establish a definitive diagnosis.

Moreover, because immune reconstitution after ART initiation can paradoxically exacerbate endobronchial lesions despite overall clinical improvement, serial bronchoscopic observation and histopathological assessment play a crucial diagnostic role.

The present case highlights the therapeutic complexity and importance of managing adverse effects during disseminated MAC treatment in patients with HIV infection. Drug-drug interactions between antiretrovirals and antimycobacterial agents require careful regimen selection. Adverse events, such as rifabutin-associated thrombocytopenia and amikacin-related ototoxicity, demand prompt recognition and adjustment. Thrombocytopenia, defined as a platelet count <50,000 cells/mm³, was observed in 5% of patients on rifabutin, compared with 4% in the placebo group in trials of MAC prophylaxis for patients with HIV [15,16]. The prescribing information for rifabutin cautions that, although the incidence was not significantly greater than that in the placebo group, rifabutin has, in rare cases, been clearly linked to thrombocytopenia [16]. While the underlying mechanism remains unclear, a potential immune-mediated mechanism has been suggested in the literature [17]. Among previously reported cases, some improved with drug discontinuation alone, whereas others required treatment with intravenous immunoglobulin and dexamethasone [17,18]. Similarly, in our case, there was little improvement after medication discontinuation, and no increase in platelet count was observed despite platelet transfusion; therefore, corticosteroid therapy was administered. Alternative etiologies for thrombocytopenia, such as advanced HIV infection, disseminated MAC itself, or bone marrow involvement, were considered but deemed less likely given the rapid recovery of the platelet count following corticosteroid initiation. The presence of positive antiplatelet antibodies and the favorable response to corticosteroid therapy strongly suggested an immune-mediated mechanism of platelet destruction. While we acknowledge that specialized drug-dependent antiplatelet antibody testing was not performed and a drug re-challenge was withheld for safety reasons, the clinical course supports a diagnosis of probable rifabutin-associated immune thrombocytopenia.

Finally, the optimal duration of treatment for disseminated MAC infection with severe endobronchial lesions in patients with HIV remains unclear. According to the NIH guidelines [2], discontinuation of maintenance therapy for disseminated MAC can be considered after at least 12 months of treatment if the patient is asymptomatic and sustains a CD4+ count >100 cells/µL for ≥6 months. In our case, at the 12-month time point, the patient met the criteria of time and CD4+ count. However, his sputum culture had just turned negative. We acknowledge that the ATS/IDSA guidelines for pulmonary MAC exclude HIV-infected patients. However, these guidelines show an important microbiological principle: long-term consolidation therapy (typically 12 months after culture conversion) is necessary to eradicate MAC in the airway. Our patient had a high mycobacterial burden initially, and the airway narrowing likely impaired localized clearance. Therefore, we considered that stopping the therapy immediately after culture conversion carried a very high risk of localized relapse. To ensure long-term microbiological safety, we applied this principle and continued the multidrug regimen for approximately two years (providing an additional 12 months of consolidation therapy) until his transfer. These challenges emphasize the need for vigilant monitoring and individualized management of complex opportunistic infections.

## Conclusions

Disseminated MAC infection rarely manifests as endobronchial polypoid lesions in patients with AIDS following the initiation of ART. This presentation is most likely consistent with a manifestation of paradoxical IRIS. In the present case, the clinical course likely reflected a dual process: initial corticosteroid therapy may have exacerbated MAC proliferation, subsequently providing a high antigen burden that triggered a profound IRIS-related inflammatory response upon immune recovery. Moreover, granulomatous inflammation in these lesions often shows high FDG uptake on PET-CT, which can closely mimic malignancy. Therefore, bronchoscopic evaluation with histopathological confirmation is essential for an accurate diagnosis. Finally, because clinical improvement may paradoxically coincide with the development of airway involvement and severe treatment-limiting adverse events, such as the rifabutin-induced immune thrombocytopenia and amikacin-related ototoxicity observed in this case, vigilant, individualized long-term management is required to balance effective infection control with minimization of drug toxicity.
